# Annotations

**Published:** 1892-02-06

**Authors:** 


### Annotations.

Hospitals and other charitable institutions have lost a loyal friend in the great Nonconformist preacher, who has just passed away. At the last meeting of the Council of the Hospital Sunday Fund held at the Mansion House, the Rev. Dr. Rigg spoke of the great loss which that fund and London generally had sustained in Mr. Spurgeon's death. Dr. Rigg moved, and the Chief Rabbi seconded, a vote of condolence with Mrs. Spurgeon and the other members of the family in their great bereavement. Mr. Burdett said it would be in the recollection of the Council that at a time when the subscriptions to the Fund were falling off, Mr. Spurgeon, at great personal inconvenience, came forward, and with his powerful voice helped them to restore the sinking balance. Mr. Spurgeon was an Englishman of the middle class; that class whose integrity, energy, and practical capacity have carried the English name and English influence into every part of the world. His creed, in the judgment of the majority of his cultivated fellow-countrymen, was a wonder and an astonishment. So far as he was concerned modern science, with its far-reaching results, might have had no existence. In theory Mr. Spurgeon was on a level with the fatalists; in practice he wrought and taught and laboured as if the whole well-being of both the present and the future world depended upon himself. His life presents an instructive story to those who appear to think that a complete scheme of the universe is open to their view. In spite of his wonderful theological position, Mr. Spurgeon contrived so to live that every intelligent Englishman respected him, and those who knew him best admired and loved him. Science lays hold of such a life, and holds it up to the religious dogmatist as a protest against his doctrine of finality. If Mr. Spurgeon, thinking what he thought, could be what he was, why may it not be possible that other holders of practically incredible doctrines may be as just, as charitable, and as effectually virtuous in all the departments of practical life as he was? The great preacher himself would have been the first to denounce what he would have called such dangerous latitudinarianism as is here taught. But greater thinkers than he was perceive clearly that no individual human mind can grasp absolute truth in its entirety. "We know in part, and we prophesy in part"; and if we could but realise this to ourselves much injurious strife would come to an end. Mr. Spurgeon's theories contrasted with Mr. Spurgeon's practice may well give us all pause, and make us all see that the very best of human beings may sometimes appear to higher intelligences exceedingly contradictory persons.

**Mr. Spurgeon.**

The *Cork Constitution* records an incident that has just taken place at the South Infirmary and County Hospital of Cork, which is not so much a case of religious intolerance as of religious foolishness. The Trustees of the hospital recently met to elect a house surgeon, in succession to Dr. N. Runciman, resigned. Two candidates were nominated from among those who applied, both legally qualified practitioners, and both probably quite suitable for the vacant post. But one of them, Dr. Jackson, seems to have had claims which specially commended him to the favour of the governing body. Among other recommendations, Dr. Jackson had served his apprenticeship at the hospital, and moreover had for some time discharged the duties of house surgeon there in the temporary absence of Dr. Runciman. He had, in one capacity or another, been connected with the institution for four years, and during that time he had gained the fullest confidence and approval of the trustees, the medical staff, the patients, and all associated with the hospital. That being so, and the other candidate having no equal claims to advance, Dr. Jackson had a considerable majority of votes among the Governors, and would indeed have been elected unanimously, but for the fact that he happened to be a Protestant. On the ground of Protestantism, and of Protestantism alone, the Rev. Canon Maguire put forward an opposition and Catholic candidate, and insisted, as a matter of right, that his candidate should be elected because "nine-tenths of the patients in the South Infirmary are Roman Catholics." There is a certain appearance of plausibility about Canon Maguire's contention, but it is only an appearance. No sensible person would choose a doctor on account of his religion any more than he would choose a clergyman on account of his homœopathy, allopathy, hydropathy, or pantopathy. Science knows nothing of religious differences; neither does medicine. We have always contended in these pages that hospital appointments, whether those of medical men, matrons, nurses, or other officials, should be made on entirely other grounds than those of religious peculiarity. In the recent dispute connected with the Matron, a Roman Catholic, of the Norfolk and Norwich Hospital, this paper ranged itself steadily on the side of the Matron, holding that religious disabilities were both wrong and foolish in themselves, and particularly out of place in hospitals and charitable institutions. Will it ever be possible for theological persons to learn what charity means? Might one not ask also for the exhibition of a small modicum of common sense?

**Religious Foolishness.**

The question whether or not ladies should take to smoking seems to have a great fascination for some of the daily papers. In view of possible conversions to the fair smokers' ranks it may be well to set before lady readers an authoritative scientific dictum on the physiological action of tobacco on the human system. According to an eminent authority, tobacco as used by us is prepared from the dried leaves of the plant *Nicotiana Tabacum*. The plant is a viscid herb, with a branched and fibrous root, and a stem from three to six feet high. The dried leaves are the parts used for smoking; but it is an ominous fact that for these there is not known any trustworthy purity test. Tobacco contains among other constituents, a liquid alkaloid, nicotina, and a concrete volatile oil Nicotianin. So small a dose as thirty grains of tobacco, administered internally have been known to cause death. "Tobacco," says the authority we are quoting from, "acts as an acro-narcotic poison, causing extreme nausea, and often purging, with utter prostration of muscular power. The heart's action is greatly reduced; the pulse becomes small weak, fluttering, and almost imperceptible; the face is pale and the extremities are cold; the pupils are dilated, the vision is impaired, the respiration laboured and the entire body bathed in a cold clammy sweat; there is great anxiety, tremour of the muscles, and a tendency to syncope; paralysis, with occasional convulsive movements and stupor which may lead to death. The habit of tobacco smoking in some individuals, especially when largely indulged in, leads to functional irregularity of the heart, weakening of the cardiac action, and disturbances of vision." The writer then goes on to state, with severe particularity, that "Tobacco is not often used medicinally, in consequence of its violent action." It is well that ladies should thoroughly understand the kind of substance they are playing with when they smoke, just as it is desirable that snake charmers should know what particular snakes are poisonous and deadly. We do not say that ladies should never smoke, but we do say that they should know what they are doing when they commence the practice. The physiological actions of tobacco which we have named have all been ascertained by experimentation on the stronger sex. It may be taken for granted that all those actions will be exerted in a decidedly more marked, unpleasant, and dangerous degree on the weaker. Two or three weeks ago we expressed the conviction that women ought not to smoke except under medical advice. Those who have appreciated the remarks of the eminent medico physiological authority we have quoted will be inclined to agree that our advice then given was not without scientific justification.

